# First identification and molecular subtyping of *Blastocystis* sp. in zoo animals in southwestern China

**DOI:** 10.1186/s13071-020-04515-2

**Published:** 2021-01-06

**Authors:** Lei Deng, Jingxin Yao, Shanyu Chen, Tingmei He, Yijun Chai, Ziyao Zhou, Xiaogang Shi, Haifeng Liu, Zhijun Zhong, Hualin Fu, Guangneng Peng

**Affiliations:** 1 The Key Laboratory of Animal Disease and Human Health of Sichuan Province, College of Veterinary Medicine, Sichuan Agricultural University, Chengdu, 611130 Sichuan People’s Republic of China; 2 Sichuan Wolong National Natural Reserve Administration, Aba, 623006 Sichuan People’s Republic of China

**Keywords:** *Blastocystis* sp., Captive wildlife, Subtypes, Phylogenetic analysis, China

## Abstract

**Background:**

*Blastocystis* sp. is an anaerobic protozoan that parasitizes many animal hosts and the human gastrointestinal tract, and its pathogenicity is controversial. Captive wildlife may be potential reservoirs for human infection with *Blastocystis* sp. The present study was performed to investigate the prevalence and subtype distribution of *Blastocystis* sp. in zoo animals in Sichuan Province, southwestern China.

**Methods:**

A total of 420 fresh fecal samples were collected from 54 captive wildlife species in four zoos in southwestern China between June 2017 and September 2019. The prevalence and subtype (ST) genetic characteristics of *Blastocystis* sp. were determined by PCR amplification of the barcode region of the *SSU* rRNA gene and phylogenetic analysis.

**Results:**

Overall, 15.7% (66/420) of the animal samples and 20.7% (14/54) of the species tested were shown to be infected with *Blastocystis* sp. The highest prevalence of *Blastocystis* sp. was found in Panzhihua Zoo (24.3%), which was significantly higher than that in Chengdu Zoo (6.9%), and Xichang Zoo (2.9%) (*P* < 0.05). There are also significant differences in the prevalence of *Blastocystis* sp. among different species (*P* < 0.05), and the highest of *Blastocystis* sp. prevalence was observed in white-cheeked gibbon, black great squirrel, and red giant flying squirrel (100%). Subtype analysis of *Blastocystis* sp. revealed nine subtypes, including six zoonotic STs (ST1-5, and ST8) and three animal-specific STs (ST10, ST14, and ST17), with ST17 as the predominant subtype (26/66) in *Blastocystis* sp.-positive isolates.

**Conclusions:**

To our knowledge, this is the first report on the prevalence and subtype distribution of *Blastocystis* sp. among captive wildlife in zoos in southwestern China. This study highlights that these animals may serve as reservoirs for human *Blastocystis* sp. infections. 
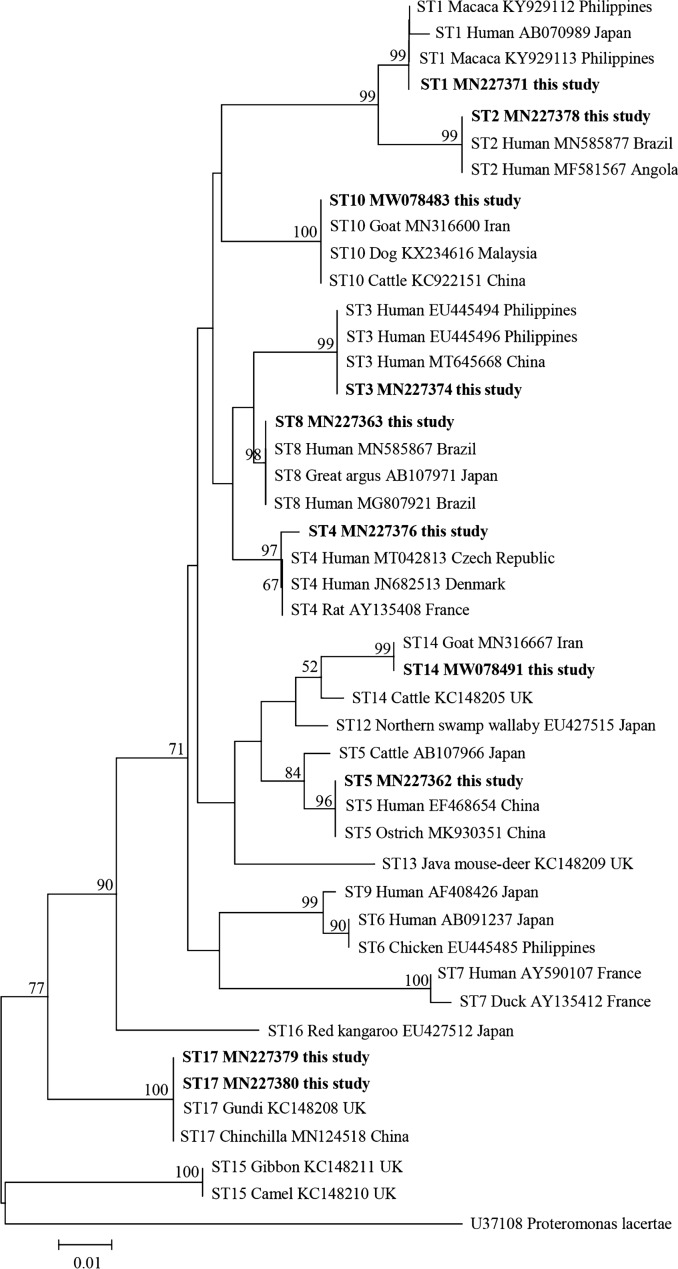

## Background

*Blastocystis* sp., belonging to the phylum stramenopiles, is a common anaerobic eukaryotic protist that inhabits the gastrointestinal tract of a wide range of hosts, including humans. It is estimated that this parasite has colonized 1 to 2 billion people worldwide based on epidemiological surveys [1]. *Blastocystis* sp. is mainly transmitted through the fecal-oral route, food, and water [2–4]. There is supporting evidence that some human infections may be caused by zoonotic transmission of *Blastocystis* sp. [5, 6].

Based on polymorphisms of small subunit (*SSU*) gene of *Blastocystis* sp., 22 subtypes (STs) consisting of ST1 to ST17, ST21, and ST23 to ST26 have been identified in humans and domestic and wild animals worldwide [7]. ST1-9 and -12 are able to infect humans, with ST1-4 being the most common, accounting for more than 90% of human *Blastocystis* sp. infections [8]. Interestingly, the prevalence of different STs among countries and among regions within the same country seems to vary greatly [9]. Remarkable genetic diversity was observed among various STs [10], and different STs exhibit diverse biological features, such as drug resistance, pathogenicity, and effects on microbiota [11–13].

Although *Blastocystis* sp. has been reported > 100 years, the clinical significance of this common parasite remains controversial [14]. Accumulating evidence shows *Blastocystis* sp. long-term colonization in asymptomatic carriers, accompanied with a healthy gut microbiota [15, 16], suggesting that it should be regarded as a member of the normal intestinal microbiota. *Blastocystis* sp. has been found in patients with irritable bowel syndrome (IBS) and inflammatory bowel disease (IBD), [17, 18], but not the presence of the protist that causes them. *In vitro* experiments using cell lines have also determined the potential pathogenicity of some specific STs of *Blastocystis* sp., such as disrupting the epithelial barrier [19] by increasing the epithelial permeability [20, 21]. Moreover, experimental infections with *Blastocystis* sp. in mouse models have shown that it can decrease the abundance of beneficial bacteria *Bifidobacterium* and *Lactobacillus* [12].

*Blastocystis* sp. has been reported in a substantial number of animal hosts, including livestock, companion animals, and captive wildlife, with greatly varying prevalence [22–24]. In recent years, several important intestinal zoonotic pathogens (e.g., *Cryptosporidium*, *Giardia*, and Microsporidia) have been reported in captive wildlife in China [25, 26], highlighting wildlife may be potential reservoirs for human to contract these infectious agents. However, less information is currently available regarding the prevalence and subtype distribution of *Blastocystis* sp. in zoo animals in China [27]. The purpose of the present study was thus to determine the genetic characteristics and subtype distribution of *Blastocystis* sp. in various zoo animals in southwestern China to better assess its zoonotic potential.

## Methods

### Sample collection

**Fig. 1:**
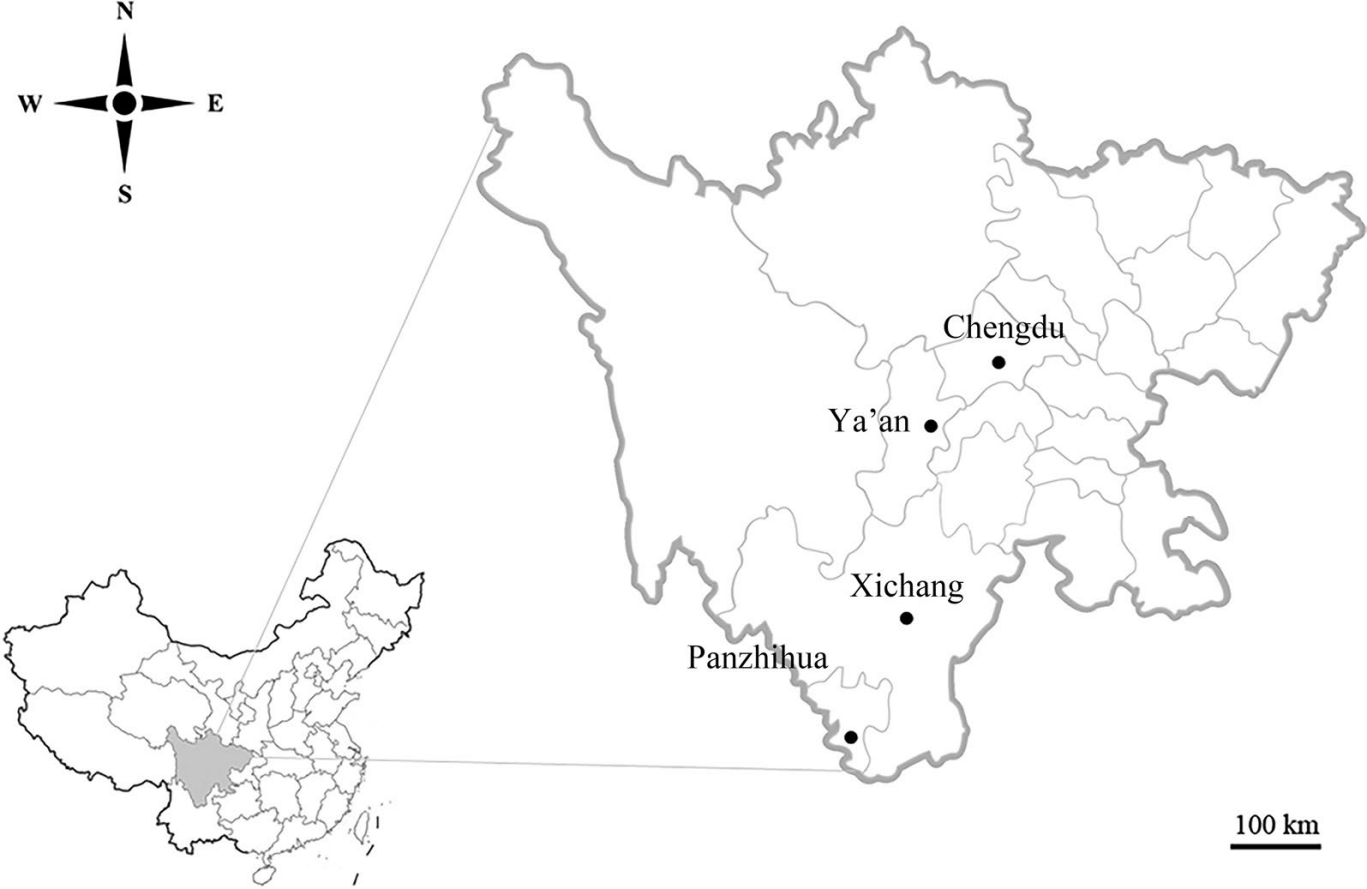
Geographical distribution of the sampled cities (filled circle) in Sichuan Province, Southwestern China

A total of 420 fresh fecal samples were collected from wildlife in Chengdu Zoo (*n* = 144), Ya’an Zoo (*n* = 204), Xichang Zoo (*n* = 35), and Panzhihua Zoo (*n* = 37) between June 2017 and September 2019 in Sichuan Province, southwestern China (Fig. 1). The collected samples include a large variety of mammalian groups and several avian species. The animals were either housed individually or in monospecific groups of 5–10 individuals sharing the same enclosures. For those animals housed individually, only one sample was collected per animal. In group housing, between two and five samples were collected, each of which was considered as individual sample. All the fresh fecal samples were collected by zookeepers before the cleaning of animal cages in the morning and were strictly controlled to minimize potential contamination between animal species. Feces samples from some avian species were collected carefully directly on the ground or in their nests. All fecal samples were collected in sterilized plastic containers using disposable sterile gloves and preserved at 4 ℃ until DNA extraction.

### DNA extraction

Fecal specimens were sieved and washed three times with distilled water by centrifugation at 3000*g* for 5 min. Genomic DNA was extracted using a QIAamp DNA

Stool Mini Kit (Qiagen, Hilden, Germany) according to the manufacturer’s instructions. DNA quality was verified by NanoDrop (Thermo Fisher Scientific, Carlsbad, CA, USA) measurements. DNA was eluted in 50 μl of nuclease-free water and stored at − 20 °C until use.

### PCR amplification

All extracted DNA was screened for the presence of *Blastocystis* sp. by a polymerase chain reaction (PCR) amplification of the barcode region of the *SSU* rRNA gene, using primers RD5 (5′-ATCTGGTTGATCCTGCCAGT-3′) and BhRDr (5′-GAGCTTTTTAACTGCAACAACG-3′) [28]. The PCR mixture (25 μl) contained 12.5 μl Taq PCR Master Mix (Sangon Biotech Co., Ltd., Shanghai, China), 1 μl each primer (0.4 μM), 2 μl genomic DNA, 1.5 mM MgCl_2_, and nuclease-free water up to desired volume. The PCR was started at 94 °C for 4 min followed by 30 cycles of 95 °C for 15 s, 60 °C for 15 s, and 72 °C for 30 s, with an extension at 72 °C for 5 min. Positive and negative controls were included in all the PCR tests. PCR products were subjected to 1.5% agarose gel (AddGene, Watertown, MA, USA) electrophoresis and visualized by staining with SYBR Safe DNA Gel Stain (Thermo Fisher Scientific).

### Sequencing and phylogenetic analysis

PCR products with the expected size (∼ 600 bp) were excised from the agarose gel and purified using a QIAquick Gel Extraction Kit (Qiagen) according to the manufacturer’s instructions. Purified products were directly sequenced on an ABI PRISMTM 3730 DNA Analyzer (Applied Biosystems, USA), using a BigDye Terminator v3.1 Cycle Sequencing kit (Applied Biosystems, Foster, CA, USA).

Nucleotide sequences obtained in the present study were subjected to BLAST searches (http://www.ncbi.nlm.nih.gov/blast/), and the reference sequences were downloaded from the GenBank database. *Blastocystis* sp. subtypes were identified by BLAST searches (http://blast.ncbi.nlm.nih.gov/Blast.cgi), and the alleles were identified at the *Blastocystis* sp. database (http://pubmlst.org/blastocystis). The sequences obtained in this study and reference sequences were aligned using the MUSCLE algorithm of MEGA-X [29]. The ambiguous positions were removed from the alignment, and then the alignment was trimmed using MEGA 6 (http://www.megasoftware.net/) before phylogenetic analysis. ST11 is not available in the barcode region, and ST21 and ST23-26 need further data for determination, so we did not include these subtypes in the phylogenetic analysis [7]. The phylogenetic tree was constructed with the neighbor-joining (NJ) method using the Mega 6 software. Evolutionary distances were calculated using the Kimura two-parameter model. Bootstrap analysis (with 1000 replicates) was performed to define the robustness of the findings. *Proteromonas lacertae* was used as outgroup for the phylogenetic analysis.

### Statistical analysis

The difference in *Blastocystis* sp. prevalence between different zoos and the order of animals was analyzed with the chi-square (*χ*^2^) test, using SPSS 20.0 (IBM, Chicago, IL, USA). The difference was considered statistically significant when *P* < 0.05. Ninety-five percent confidence intervals (95% CIs) and odds ratios (ORs) were also calculated to explore the strengths of association between *Blastocystis* sp. positivity and each factor.

## Results

### Prevalence of *Blastocystis* sp. in captive wild animals

It is worth noting that the prevalences herein are the minimum since we used barcoding primers rather than qPCR. In the present study, 66 of 420 (15.7%) fecal samples collected from four animal zoos in China were determined to be *Blastocystis* sp. positive by PCR amplification of the barcode region of the *SSU* rRNA gene. Specifically, 10 of 144 (6.9%) animals sampled from Chengdu Zoo, 46 of 204 (22.5%) samples from Ya’an Zoo, 1 of 35 (2.9%) samples from Xichang Zoo, and 9 of 37 (24.3%) samples collected from Panzhihua Zoo were *Blastocystis* sp. positive (Table 1). The difference in *Blastocystis* sp. prevalence was significant in four zoos (P < 0.05). The prevalence of *Blastocystis* sp. in nonhuman primates (NHPs) was 30.5%, which is higher than that in Rodentia (18.2%), Artiodactyla (12.8%), birds (8.8%), and Carnivora (5.9%). Similarly, the difference of *Blastocystis* sp. prevalence among different order animals was significant (P < 0.05).

**Table 1:** Factors associated with the prevalence of *Blastocystis* sp. in zoo animals in China

Factors	No. of examined	No. of positive	Prevalence (%) (95% CI)	OR (95% CI)	*P* value
Zoo					
Ya'an Zoo	204	46	22.5% (16.8–28.3)	0.906 (0.399–2.056)	0.813
Chengdu Zoo	144	10	6.9% (2.8–11.1)	0.232 (0.086–0.624)	0.004
Xichang Zoo	35	1	2.9% (0–8.4)	0.092 (0.011–0.767)	0.027
Panzhihua Zoo	37	9	24.3% (10.5–38.1)	Reference	
Host					
Primates	128	39	30.5% (22.5–38.4)	4.541 (2.201–9.370)	0.000
Carnivora	85	5	5.9% (0.8–10.9)	0.648 (0.217–1.936)	0.437
Rodentia	33	6	18.2% (5.0–31.3)	2.303 (0.782–6.779)	0.130
Artiodactyla	39	5	12.8% (2.3–23.3)	1.524 (0.495–4.691)	0.463
Perissodactyla	4	0	0		
Diprotodontia	3	0	0		
Erinaceidae	3	0	0		
Birds	125	11	8.8% (3.8–13.8)	Reference	
Total	420	66	15.7% (12.2–19.2)		

In general, of the 54 species tested in this study, 14 (20.7%) were positive for *Blastocystis* sp. (Table 2). Specifically, of the 29 species tested at the Chengdu Zoo, 6 (20.7%) were positive for *Blastocystis* sp. At the Ya’an Zoo, the prevalence of the parasite was 33.3% (5/15) among the species screened; 7.7% (1/13) and 33.3% (3/9) species at Xichang Zoo and Panzhihua Zoo in the present study were shown to be infected with *Blastocystis* sp. respectively.

**Table 2:** Animal samples collected from various hosts from four different zoos in Sichuan Province, southwestern China

Host	Scientific name	No. of examined	YA	CD	XC	PZH	No. of *Blastocystis*-positive
Primates							
De Brazza's monkey	*Cercopithecus neglectus*	1	1				0
Rhesus macaque	*Macaca mulatta*	17	15	2			10
Ring-tailed lemur	*Lemur catta*	13	13				6
Squirrel monkey	*Saimiri sciureus*	93	93				19
White-cheeked gibbon	*Hylobates leucogenys*	4	4				4
Carnivora							
African lion	*Panthera leo*	14	14				0
Bengal tiger	*Panthera tigris*	2	1			1	0
Leopard	*Panthera pardus*	3	3				2
Lynx	*Lynx lynx*	1	1				0
Asiatic black bear	*Ursus thibetanus*	12				12	3
Brown bear	*Ursus arctos*	1				1	0
Asiatic wild dog	*Cuon alpinus*	2	2				0
Arctic fox	*Vulpes lagopus*	3		3			0
Wolf	*Canis lupus Linnaeus*	2		2			0
Raccoon dog	*Nyctereutes procyonoides*	2		2			0
Eurasian badger	*Meles meles*	2		2			0
Eurasian river otter	*Lutra lutra*	2		2			0
Ferret	*Mustela pulourius furo*	2		2			0
Red panda	*Ailurus fulgens*	17	17				0
Raccoon	*Procyon lotor*	18	13	5			0
Coati	*Nasuella olivacea*	1		1			0
Civet	Paguma sp.	1		1			0
Rodentia							
Chipmunk	Tamias sp.	5		5			0
Prairie dogs	Cynomys sp.	3		3			0
Polatouche	*Pteromys volans*	2		2			0
Black great squirrel	*Ratufa bicolor*	1		1			1
Red giant flying squirrel	*Petaurista petaurista*	1		1			1
Capybara	*Hydrochoerus hydrochaeris*	4	4				0
Guinea pig	*Cavia porcellus*	2		2			0
Chinchilla	*Chinchilla lanigera*	6		6			4
Beaver	*Castor fiber*	4		4			0
Nepal porcupine	*Hystrix brachyura subcristata*	3		3			0
Hamster	Cricetulus sp.	2		2			0
Artiodactyla							
Sika deer	*Cervus nippon*	11		3	3	5	1
Red muntjac	*Muntiacus muntjak*	7		7			0
Fallow deer	*Dama dama*	3		3			0
Roe deer	*Capreolus pygargus*	1		1			0
Alpaca	*Vicugna pacos*	11	11				4
Two-humped camel	*Camelus bactrianus*	4			2	2	0
Yellow cattle	*Bos taurus domestica*	2			2		0
Perissodactyla							
Horse	*Equus caballus*	3			1	2	0
Common zebra	*Equus burchellii*	1				1	0
Diprotodontia							
Parma wallaby	*Macropus parma*	3		3			0
Erinaceidae							
Hedgehog	*Erinaceus amurensis*	3		3			0
Galliformes							
Green peafowl	*Pavo muticus*	12			5	7	1
Chicken	*Gallus gallus*	51		41	10		0
Ring-necked Pheasant	*Phasianus colchicus*	1			1		0
Guinea fowl	Numididae	3			3		0
Struthionformes							
Common ostrich	*Struthio camelus*	19	12		1	6	6
Psittaciformes							
Green-winged macaw	*Ara chloroptera*	1			1		0
Columbiformes							
Common pigeon	*Columba livia*	34		31	3		4
Anseriformes							
Goose	*Anser cygnoides domesticus*	2			2		0
Strigiformes							
Eurasian eagle owl	*Bubo bubo*	1			1		0
Ratitae							
Cassowary	Casuarius sp.	1		1			0
Total		420	204	144	35	37	66

YA = Ya’an Zoo; CD = Chengdu Zoo; XC = Xichang Zoo; PZH = Panzhihua Zoo

Interestingly, the prevalence of *Blastocystis* sp. varies greatly among different species (Table 3). The highest *Blastocystis* sp. prevalence was observed in white-cheeked gibbon (100%, 4/4). By comparison, sika deer and green peafowl showed lower *Blastocystis* sp. prevalence, accounting for 9.1% and 8.3% respectively.

**Table 3:** Prevalence of *Blastocystis* sp. among different species

Species	No. of examined	No. of positive	Prevalence (%)	YA	CD	XC	PZH
Squirrel monkey	93	19	20.4	ST17 (19)			
Rhesus macaque	17	10	58.8	ST1 (10)			
White-cheeked gibbon	4	4	100	ST2 (3); ST3 (1)			
Ring-tailed lemur	13	6	46.2	ST1 (3); ST2 (3)			
Chinchilla	6	4	66.7		ST17 (4)		
Red giant flying squirrel	1	1	100		ST4 (1)		
Black great squirrel	1	1	100		ST4 (1)		
Alpaca	11	4	36.4	ST10 (2); ST14 (2)			
Sika deer	11	1	9.1				ST1 (1)
Asiatic black bear	12	3	25				ST17 (3)
Leopard	3	2	66.7	ST1 (1); ST5 (1)			
Common pigeon	34	4	11.8		ST8 (4)		
Green peafowl	12	1	8.3			ST3 (1)	
Common ostrich	19	6	31.6	ST5 (1)			ST5 (5)
Total	237	66	27.8	ST17 (19); ST1 (14); ST2 (6); ST5 (2); ST10 (2); ST14 (2); ST3 (1)	ST8 (4); ST17 (4); ST4 (2)	ST3 (1)	ST5 (5); ST17 (3); ST1 (1)

YA = Ya’an Zoo; CD = Chengdu Zoo; XC represents Xichang Zoo; PZH = Panzhihua Zoo

### Subtype distributions of *Blastocystis* sp. in captive wild animals

Among the 66 *Blastocystis* sp.-positive samples, 9 subtypes were identified, including 6 zoonotic STs (ST1-5, and ST8) and 3 animal-specific STs (ST10, ST14, and ST17). ST17 (allele 160) (39.4%, 26/66) was the dominant subtype in zoo animals examined in the present study (Table 4), followed by ST1 (allele 1) (22.7%, 15/66), ST5 (allele 118) (10.6%, 7/66), ST2 (allele 15) (9.1%, 6/66), and ST8 (allele 21) (6.1%, 4/66). ST3 (allele 34), ST4 (allele 42), ST10 (allele 152), and ST14 (allele 157) were only found in two fecal samples respectively (Table 4). Notably, ST1 has the widest host range in zoo animals, detected in rhesus macaque, ring-tailed lemur, leopard, and sika deer (Table 4). Meanwhile, ST17 was identified in four species of animals, including squirrel monkey, Asiatic black bear, and chinchilla (Table 4).

**Table 4:** Subtype distributions from different animal species

Host	*Blastocystis* sp. STs									Sequences
	1	2	3	4	5	8	10	14	17	
Primates										
Rhesus macaque	10									10
Ring-tailed lemur	3	3								6
Squirrel monkey									19	19
White-cheeked gibbon		3	1							4
Carnivora										
Leopard	1				1					2
Asiatic black bear									3	3
Rodentia										
Chinchilla									4	4
Red giant flying squirrel				1						1
Black great squirrel				1						1
Artiodactyla										
Sika deer	1									1
Alpaca							2	2		4
Birds										
Common ostrich					6					6
Common pigeon						4				4
Green peafowl			1							1
Total	15	6	2	2	7	4	2	2	26	66

### Genetic characteristics of *Blastocystis* sp. subtypes

The identity analysis of the *SSU* rRNA gene revealed that 15 sequences of ST1 isolates identified in NHPs, leopard, and sika deer were identical to those from Philippine long-tailed macaque in the Philippines (KY929113). Similarly, six ST2 sequences from NHPs (ring-tailed lemur, and white-cheeked gibbon) showed 100% identity with GenBank sequences MN585877 (from human in Brazil) and MF581567 (from human in Angola). ST3 and ST4 sequences had the largest identity (99.62% and 98.97%) related to that from human in China (MT645668), and human in Czech Republic (MT042813), with two and six nucleotide substitutions respectively. Meanwhile, one leopard and six ostrich-derived ST5 sequences had 98.95% identity with that from an ostrich in China (MK930351), with six single-nucleotide polymorphisms (SNPs). In terms of four pigeon-derived ST8 isolates, the sequences had 99.65% identity with that from human in Brazil (MN585867), with two nucleotide substitutions being observed. The sequences of ST10 and ST14 from alpaca were identical to the GenBank sequences MN316600 and MN316667, both from goat in Iran respectively. For 26 ST17 isolates, two representative sequences were obtained from NHPs, Asiatic black bear, and chinchillas. The sequence (MN227379) of ST17 isolates showed 100% identity to the sequence of ST17 isolated from a gundi in Libya (KC148208). The sequence (MN227380) exhibited 99.15% identity to the sequence of ST17 isolated from a chinchilla in China (MN124518), with five SNPs.

### Phylogenetic analysis of *Blastocystis* sp.

**Fig. 2:**
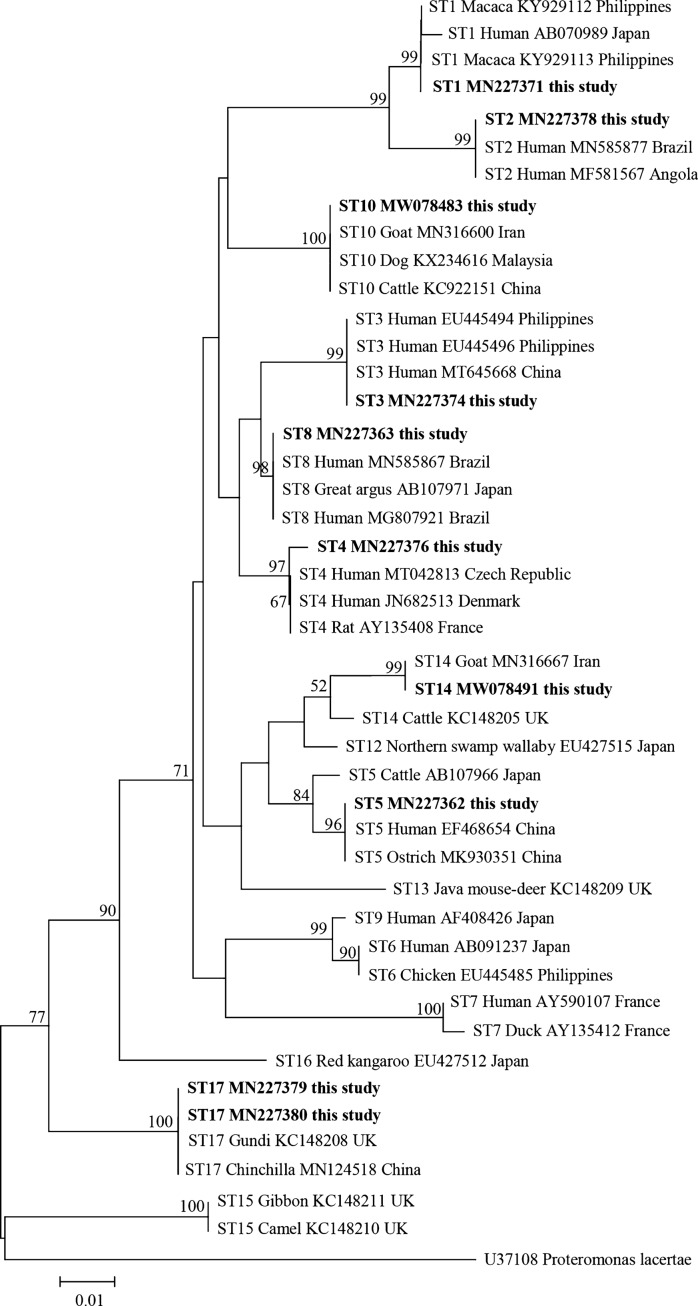
Phylogenetic relationships among nucleotide sequences of barcode regions of small subunit ribosomal RNA (*SSU* rRNA) of *Blastocystis* sp. The neighbor-joining method was used to construct the trees by the Kimura-2-parameter model. The numbers on the branches are percent bootstrapping values from 1000 replicates, with values > 50% shown in the tree. Each sequence is identified by its subtypes, hosts, accession number, and country. *Blastocystis* subtypes identified in the present study are indicated in bold type

A total of 10 representative sequences were obtained from 66 *Blastocystis* sp. isolates in the present study. The sequences obtained in this study showed high identity with the reference sequences of *Blastocystis* sp. in GenBank. Newly acquired sequences belong to ST1, ST2, ST3, ST4, ST5, ST8, ST10, ST14, and ST17. ST1 and ST2 along with sequences originating from humans and Macaca clustered together. ST3 and ST8 grouped together with sequences mainly from humans. ST4 clustered together with sequences from rats and humans. ST14 along with sequences isolated from goat and cattle grouped together. ST5 along with sequences isolated from ostrich, cattle, and human clustered together. ST10 formed a clade with sequences from dog, cattle, and goat. Similarly, ST17 formed a separate branch with sequences from gundi and chinchilla (Fig. 2).

## Discussion

*Blastocystis* sp. is a common intestinal protozoan parasite with uncertain pathogenicity. It is believed that zoonotic STs often spread between animals and humans, as some STs of animal origin are a large potential reservoir for human infections [30–32]. *Blastocystis* sp. has been identified in many animal hosts, such as NHPs, pigs, cattle, sheep, goats, dogs, birds, and various captive wildlife [23, 24, 33]. The prevalence of *Blastocystis* sp. in zoo animals examined in this study was 15.7% (66/420), which was lower than that in captive wild animals in Qinling Mountains, China (40.2%, 200/497) [34], in zoo animals in the UK (35.5%, 82/231) [35], in zoo animals in Japan (39.0%, 46/118) [36], in various captive animals in France (32.2%, 99/307) [37], and in zoo animals in Western Australia (42%, 32/76) [5], while the *Blastocystis* sp. prevalence in the present study was higher than that in zoo animals in three cities in China (6.0%, 27/450) [27]. However, it is difficult to explain the discrepancy of *Blastocystis* sp. prevalence between different countries or within the same country because many factors contribute to the effect the prevalence, such as the size of examined samples, animal species, or management methods.

The prevalence of *Blastocystis* sp. among the four zoos was significantly different in the present study. The prevalence of *Blastocystis* sp. in Panzhihua Zoo was the highest (24.3%), which was significantly higher than that in Chengdu Zoo (6.9%) and Xichang Zoo (2.9%) (*P* < 0.05). The difference in the prevalence of *Blastocystis* sp. may be related to the zoo’s sanitary conditions and management methods. The sanitary conditions are relatively poor, and there is no good deworming and immunization program in the Panzhihua Zoo. In addition, the host and number of samples collected in each zoo are different, which may also be one of the reasons for the difference in *Blastocystis* sp. prevalences.

In the present study, 9 *Blastocystis* STs, including ST1-5, ST8, ST10, ST14, and ST17, were identified in 66 *Blastocystis* sp.-positive samples from captive wildlife. Of them, four subtypes (ST1-3, and ST17) were identified in NHPs (rhesus macaque, ring-tailed lemur, squirrel monkey, and white-cheeked gibbon). Generally, ST1-3 has been frequently determined in NHPs, while ST17 was identified to a lesser extent [34, 37]. Interestingly, a more recent study showed zoo animals and staff were infected with ST1-3 and showed high consistency with known sequences from NHPs and humans, highlighting that these STs have zoonotic potential [38]. In China, ST1, ST2, ST3, ST4, ST5, ST9, ST10, and ST13 were identified in NHPs [27, 31, 39, 40], with ST1 and ST2 being the most common. In this study, *Blastocystis* sp. was first discovered in squirrel monkey, and subtype analysis showed all isolates from squirrel monkey were ST17.

To date, several zoonotic STs have been identified in Carnivora, such as ST1-5, ST7-8, and ST10 [32, 33, 38]. In the current study, sequences obtained from Carnivora belonged to ST1, ST5, and ST17. These isolates were infecting the leopard (ST1 and ST5) and Asiatic black bear (ST17). As previously reported, the animals belonging to the order of Carnivora such as South American coati, Arctic fox, and dogs were determined to be infected with ST1 [38, 41]. Hussein *et al.* reported that ST1 inoculated into Wistar rats could cause moderate and severe degrees of pathological changes, suggesting the potential pathogenicity of this subtype [42]. ST5 was the most predominant subtype in pigs [43], but it was also identified in a various animals, such as NHPs, cattle, sheep, rodents, and birds [23]. However, ST5 was rarely found in carnivores, and only a few wild carnivores, such as Northern tiger cat, and meerkat were determined to have ST5 infection [27, 38]. In addition, ST5 infection has also been reported in stray and domestic dogs in India [44] and the Philippines [45]. ST17 has been identified in gundi in Libya [46] and in cattle in the USA [47]. While there is no study on ST17 infections in Carnivora, this is the first report of ST17 infection in Asiatic black bear in China, demonstrating a wider host range of this subtype.

Previous studies reported many animals in the order of Artiodactyla harboring the *Blastocystis* sp., such as pigs, cattle, sheep, goats, camels, and deer [23, 33]. The majority of STs (ST1-7, ST10, ST13-15, and ST17) have been identified in Artiodactyla to date [46, 48]. Among them, ST10 was the most common subtype in cattle in the US [49], Denmark [50], and China [51, 52]. In the present study, ST1 was found in sika deer. Several studies have reported on *Blastocystis* sp. infections in deer, with different subtype distributions. For example, sika deer, fallow deer, and white-lipped deer were reported to be infected with ST10 in China [34]. ST4 and ST10 were also reported in red deer, and muntjac deer were found with ST14 infection in the UK [53]. Strikingly, a rare ST (ST13) was also determined in a mouse deer in the UK [46] and in Java mouse-deer in France [37]. The distribution of STs in alpaca in the present study was consistent with a previous study in the Qinglin Mountains in China, in which all isolates identified belonged to ST10 and ST14 [34]. Similarly, ST10 infection in alpaca was also reported in French zoos [37]. Overall, these data suggest that deer and alpaca may serve as natural hosts of *Blastocystis* sp.

In this study, ST4 and ST17 were identified in rodents, corroborating previous data on pet rodents in Sichuan Province [54]. ST4 was originally isolated from a healthy Wistar rat in Singapore [55], and rodents were proposed to be a main reservoir of ST4. Recent studies confirmed that other STs, such as ST1-3, ST5, ST7, ST8, ST10, and ST17, can also be found in rodents [46, 53]. ST4 infection has been the reported for the first time in red giant flying squirrel and black great squirrel, expanding its host range. The observation of ST17 in shrew-faced squirrels in the United Arab Emirates suggests rodents may be the natural host of this subtype [56]. In the present study, ST17 was also observed in chinchilla in China for the first time to our knowledge, indicating a novel host for this subtype.

Regarding the non-mammalian groups, birds have already been considered potential reservoirs of *Blastocystis* sp. transmission to humans [57]. It is believed that birds usually harbor ST6 and ST7, which are considered ‘avian STs’ because of their predominance in this host group. Nevertheless, of the 11 avian isolates characterized in the present study, none were identified as belonging to the “avian” ST6 or ST7. Six of them belonged to ST5, four to ST8, and one to ST3; these results are similar to those of a previous study in birds in French zoos where ST5 was the dominant subtype [37]. It should be noted that our previous study reported that ST8 was the predominant subtype in captive birds in Sichuan Province [58], suggesting that this subtype may circulate among birds in the investigated areas. However, the transmission characteristics of these zoonotic subtypes warrant further examination in future studies.

## Conclusions

The present study described the prevalence, subtype distribution and genetic characterizations of *Blastocystis* sp. for the first time in zoo animals in southwestern China. The data demonstrated that *Blastocystis* sp. could be maintained and transmitted between wildlife. Moreover, the zoonotic subtypes were identified in captive wildlife suggesting that these animals may serve as natural reservoirs for human *Blastocystis* sp. infections. The present results could provide fundamental information for the evaluation of potential zoonotic transmission between wildlife and humans.

## Data Availability

The nucleotide sequences generated in the present study have been deposited in GenBank (https://www.ncbi.nlm.nih.gov/) under accession numbers MN227371, MN227378, MN227374, MN227376, MN227362, MN227363, MW078483, MW078491, MN227379, and MN227380. The datasets used and/or analyzed during the current study are available from the corresponding author on reasonable request.

## Abbreviations

*SSU* rRNA: Small subunit ribosomal RNA
STs: Subtypes
IBS: Irritable bowel syndrome
IBD: Inflammatory bowel disease
PCR: Polymerase chain reaction
ORs: Odds ratios
NHPs: Nonhuman primates
SNPs: Single nucleotide polymorphisms
